# Correlation between clinical phenotype and electromyographic parameters in amyotrophic lateral sclerosis

**DOI:** 10.1007/s00415-022-11404-4

**Published:** 2022-10-02

**Authors:** Eleonora Colombo, Alberto Doretti, Francesco Scheveger, Alessio Maranzano, Giulia Pata, Delia Gagliardi, Megi Meneri, Stefano Messina, Federico Verde, Claudia Morelli, Stefania Corti, Luca Maderna, Vincenzo Silani, Nicola Ticozzi

**Affiliations:** 1 Department of Neurology and Laboratory of Neuroscience, IRCCS Istituto Auxologico Italiano, P.le Brescia 20, 20149 Milan, Italy; 2 Neurology Residency Program, Università degli Studi di Milano, Via Festa del Perdono 7, 20122 Milan, Italy; 3 Neurology Unit, Fondazione IRCCS Ca’ Granda Ospedale Maggiore Policlinico, Via Francesco Sforza 35, 20122 Milan, Italy; 4 Department of Pathophysiology and Transplantation, Università degli Studi di Milano, Via Festa del Perdono 7, 20122 Milan, Italy

**Keywords:** Amyotrophic lateral sclerosis, Motor neuron disease, Electromyography, Clinical neurophysiology

## Abstract

**Introduction:**

Even if electromyography (EMG) is routinely used to confirm the diagnosis of amyotrophic lateral sclerosis (ALS), few studies have analysed the correlation between electrophysiological parameters and clinical characteristics of ALS. We assessed if the quantification of active denervation (AD) and chronic denervation (CD) provides clinicians with information about phenotype, disease progression and survival in ALS patients.

**Methods:**

We studied a cohort of 689 ALS patients recording the following parameters: age and site of onset, survival, MRC scale for muscle strength evaluation, burden of upper and lower motor signs as measured with specific scales (PUMNS and LMNS, respectively), ALSFRS-R, progression rate (ΔFS), MITOS and King’s Staging systems (KSS). We performed EMG on 11 muscles, and calculated semiquantitative AD and CD scores for each limb, as well as for the bulbar and spinal regions.

**Results:**

We found a positive correlation between AD and CD scores with LMNS (respectively *p = *4.4 × 10^–37^ and *p = *2.8 × 10^–45^) and a negative correlation with MRC (respectively *p = *4.5 × 10^–35^ and *p = *3.0 × 10^–35^). Furthermore, patients with higher spinal AD and CD scores had significantly lower ALSFRS-R scores, and higher KSS and MITOS stages. Conversely, only AD was associated to higher ΔFS (*p = *1.0 × 10^–6^) and shorter survival (*p = *1.1 × 10^–5^).

**Conclusion:**

Our results confirmed that EMG examination represents not only a diagnostic instrument, but also a prognostic tool. In this context, AD seems to be a reliable predictor of disease’s progression and survival while CD better describes functional disability.

**Supplementary Information:**

The online version contains supplementary material available at 10.1007/s00415-022-11404-4.

## Introduction

Amyotrophic lateral sclerosis (ALS) is a fatal neurodegenerative disorder of adult life of undetermined aetiology that primarily affects the motor neurons of the cerebral cortex, brainstem, and spinal cord. The El Escorial criteria for the diagnosis of ALS were initially published in 1994 and required the presence of lower motor neuron (LMN) and upper motor neuron (UMN) signs in different body regions [1]; subsequently, to increase diagnostic sensitivity, El Escorial criteria have been revised [2] recognizing the increasing importance of laboratory exams, including neurophysiological studies, to support the diagnosis. To further integrate electrophysiological criteria with clinical examination findings, an expert consensus group formulated the Awaji criteria [3] that improve, even if modestly, the sensitivity of ALS diagnosis compared to the revised El Escorial criteria [4–9]. Lastly, the Gold Coast criteria have been recently formulated with the target to further increase sensitivity and simplify the previous ones [10].

In this context, electromyography (EMG) represents the most important diagnostic test in ALS and, according to the Awaji criteria [3], neurophysiological signs of LMN dysfunction (insertional activity, positive sharp waves, fibrillation and fasciculations potentials, increased amplitude and duration of motor unit potentials, abnormal recruitment pattern) have equivalent weight to clinical LMN manifestations. Moreover, EMG allows for the identification of LMN impairment not only in weak muscles, but also in clinically non-involved regions [11]. Even if EMG is routinely used in clinical practice to confirm diagnostic suspicion of ALS, few studies have analysed correlation between electrophysiological parameters and clinical characteristics of ALS [12–20].

The aim of our work is to assess if the quantification of active denervation (AD) and chronic denervation (CD) provides clinicians with information about clinical features, disease progression and survival in ALS patients. In this context, EMG could represent not only a diagnostic test but also a tool to better define clinical phenotype and prognosis of motor neuron disease.

## Patients and methods

### Study cohort

A cohort of patients diagnosed with motor neuron disease [ALS, primary lateral sclerosis (PLS) and progressive muscular atrophy (PMA)] according to El Escorial revised criteria was recruited at IRCCS Istituto Auxologico Italiano between 2008 and 2021. The following clinical information were collected: age at onset; survival (considering tracheostomy as equivalent to death); ALSFRS-R score at evaluation; progression rate (ΔFS) calculated with the formula [(48 − ALSFRS−R score)/disease duration expressed in months]; clinical stages according to the King’s and Milano-Torino (MITOS) staging systems. With regard to motor assessment, weakness of spinal muscles was studied using the MRC scale for three muscle groups for each limb (shoulder abductors, elbow flexors, wrist dorsiflexors, hip flexors, knee extensors and ankle dorsiflexors) for a total score of 0–60. The burden of LMN involvement was assessed using LMNS, scoring weakness and wasting in each limb on a scale from 0 to 3 [21]. The scale was modified to assess the presence of LMN impairment also in thoracic and bulbar regions, assigning 1 point each, for a maximum score of 14. The Penn UMN score (PUMNS) was used to define the severity of UMN impairment in the bulbar region (score 0–4) and each limb (score 0–7), for a total score of 0–32 [22].

### Neurophysiological evaluation

EMG was performed on all patients using a standard protocol [4]. Examinations were then reviewed by a neurophysiologist with experience in the field of motor neuron diseases, blinded to clinical phenotype and severity of ALS patients, and semiquantitative scores for AD and CD were calculated for the bulbar, cervical and lumbosacral regions.

The following body regions and muscles were evaluated during score calculation: bulbar region (right genioglossus, left and right masseter), right arm (biceps brachii, first dorsal interosseus), left arm (triceps brachii, first dorsal interosseus), right lower limb (vastus medialis, tibialis anterior) and left lower limb (gastrocnemius medialis, tibialis anterior). Each muscle was investigated in three different sites and every site was explored using 3 or 4 needle insertions. For each of the five body regions a score ranging from 0 to 3 was assigned according to the amount of positive sharp waves and/or fibrillation potentials for active denervation and characteristics of Motor Unit Action Potentials (MUAPs) for chronic denervation (Supplemental Table 1). AD and CD scores for spinal muscles were then obtained by summing the partial scores for each limb.

### Statistical analysis

Statistical analysis was carried out with IBM Statistical Package for Social Science (SPSS) version 26. Descriptive statistics are reported as numbers and percentages for categorical variables or mean and standard deviation for continuous variables. Survival analysis was performed dividing patients in two groups according to the median values of spinal active and chronic denervation and building Kaplan–Meier curves. To assess the effect of denervation on survival, Cox proportional hazard regression was used. The log-rank test was used to compare survival across groups. EMG parameters were compared with continuous variables using Pearson correlation. A *p* < 0.05 was considered significant, and all tests were two-sided.

### Standard protocol approval and patients’ consent

Informed consent for using anonymized data for research purposes was obtained from all patients or their authorized legal representatives. Anonymized data are archived on Zenodo (https://doi.org/10.5281/zenodo.6818074) and will be disclosed upon reasonable request. This study was approved by the Ethics Committee of IRCCS Istituto Auxologico Italiano (DAMARE 2021_05_18) and conducted according to the principles expressed in the Declaration of Helsinki.

## Results

### Cohort description

We recruited a cohort of 689 patients, 251 (36.4%) of whom were females and 438 (63.6%) males. The mean age of disease onset was 60.4 years (± 12.2), while average survival was 37.6 months (± 36.0). Site of onset (known in 686/689 cases) was bulbar in 155 (22.6%) and spinal in 531 (77.4%) patients. The median time to visit was 21.6 months after disease onset (range 1.5–273.7). With regard to the motor phenotype, we could divide the cohort into the following phenotypes: classic ALS (*n = *367, 53.5%), flail arm (*n = *30, 4.4%), flail leg (*n = *17, 2.5%), UMN-predominant (UMNp) ALS (*n = *68, 9.9%), PLS (*n = *26, 3.8%), respiratory ALS (*n = *14, 2.0%), bulbar ALS (*n = *137, 20.0%) and PMA (*n = *27, 3.9%). ALSFRS-R and ΔFS score were available for 481 patients and the mean scores were 38.2 (± 6.9) and 0.86 (± 0.92) respectively. MITOS and KSS were available respectively for 390 and 684 patients. MRC was calculated for 574 ALS individuals with an average score of 50.0 (± 9.3) in our cohort. LMNS and PUMNS were available for 684 and 685 patients with a mean score of 4.8 (± 3.1) and 9.8 (± 7.3) respectively (Table 1).

**Table 1:** Demographic and clinical characteristics of the study cohort

	ALS cohort			
	*n*	%	Mean	Median
Sex				
M	438	63.6		
F	251	36.4		
Site of onset				
Bulbar	155	22.6		
Spinal	531	77.5		
Phenotype				
Classic	367	53.5		
Bulbar	137	20.0		
Respiratory	14	2.0		
Flail arm	30	4.4		
Flail leg	17	2.5		
PMA	27	3.9		
UMN predominant	68	9.9		
PLS	26	3.8		
Age at onset^a^			60.4	61.9
Time to NIV^b^			27.1	20.1
Time to PEG^b^			26.9	23.2
ALSFRS-R			38.2	40.0
Progression rate			0.86	0.60
Survival^b^			37.6	26.8

*NIV* non-invasive ventilation; *PEG* percutaneous endoscopic gastrostomy; *PMA* progressive muscular atrophy; *PLS* primary lateral sclerosis; *UMN* upper motor neuron

^a^Expressed in years

^b^Expressed in months

### Clinical and neurophysiological results

**Fig. 1:**
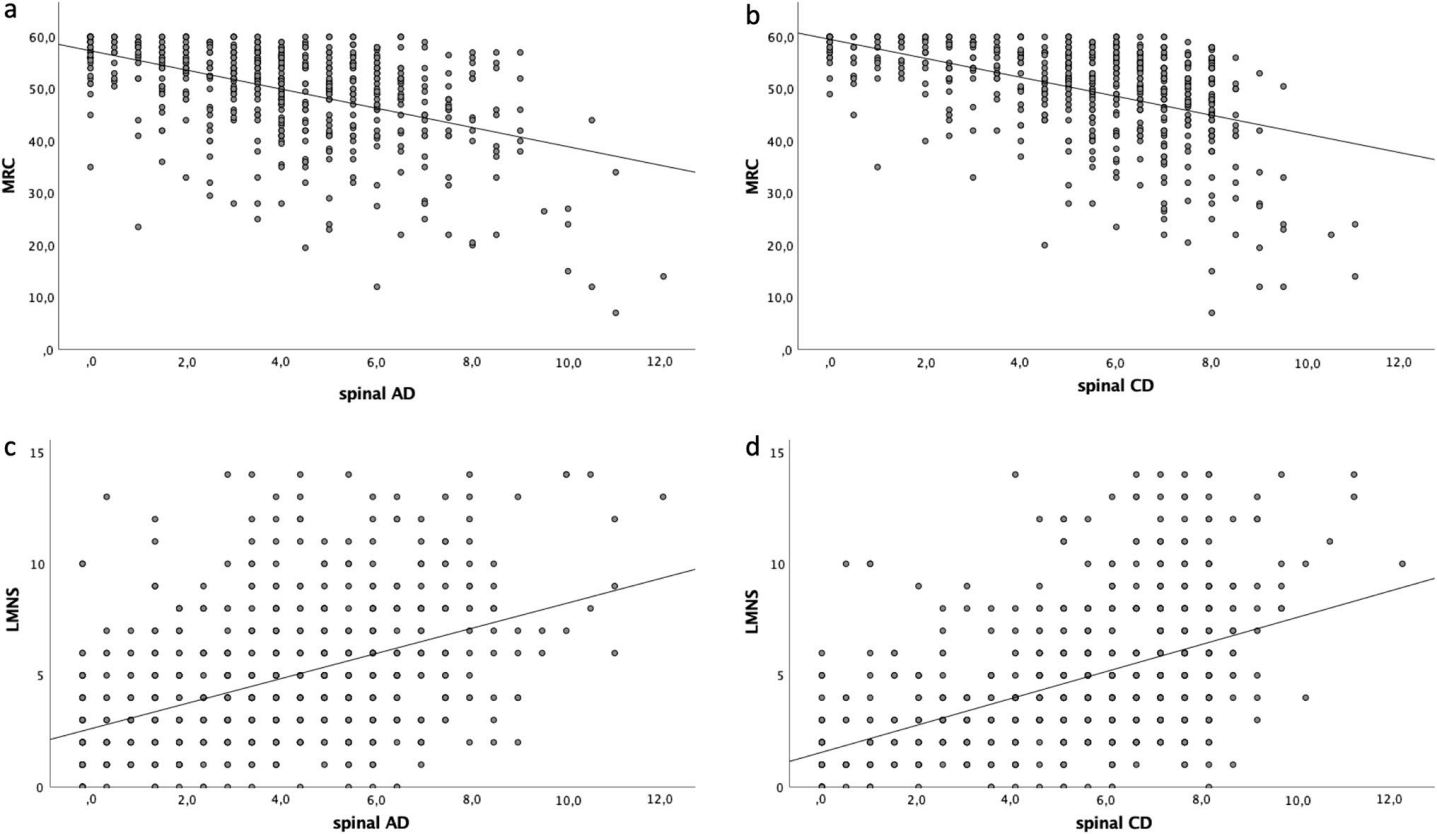
Scatter plots showing the correlation of MRC with spinal AD (*R*^2^ = 0.234, *p = *4.5 × 10^–35^) and CD (*R*^2^ = 0.236 *p = *3.0 × 10^–35^) scores (**a** and **b**) and of LMNS with spinal AD (*R*^2^ = 0.211 *p = *4.4 × 10^–37^) and CD (*R*^2^ = 0.254 *p = *2.8 × 10^–45^) scores (**c** and **d**). Each grey dot represents a single patient. Trend line is shown in black

To define if electromyographic parameters represent good markers of muscle weakness, we first compared spinal AD and CD scores with MRC and observed negative correlations with both AD (*R*^2^ = 0.234, *p = *4.5 × 10^–35^) and CD (*R*^2^ = 0.236 *p = *3.0 × 10^–35^) scores (Fig. 1a, b). Similarly, we found a positive correlation between LMNS and both AD (*R*^2^ = 0.211 *p = *4.4 × 10^–37^) and CD (*R*^2^ = 0.254 *p = *2.8 × 10^–45^) scores (Fig. 1c, d). The association between clinical and neurophysiological signs of LMN impairment confirmed that EMG parameters well describe the degree of clinical LMN dysfunction. With regard to the burden of UMN signs, we observed a negative correlation between PUMNS values and spinal AD (*R*^2^ = 0.01, *p = *0.008) and CD (*R*^2^ = 0.035, *p = *9.0 × 10^–7^) scores.

**Fig. 2:**
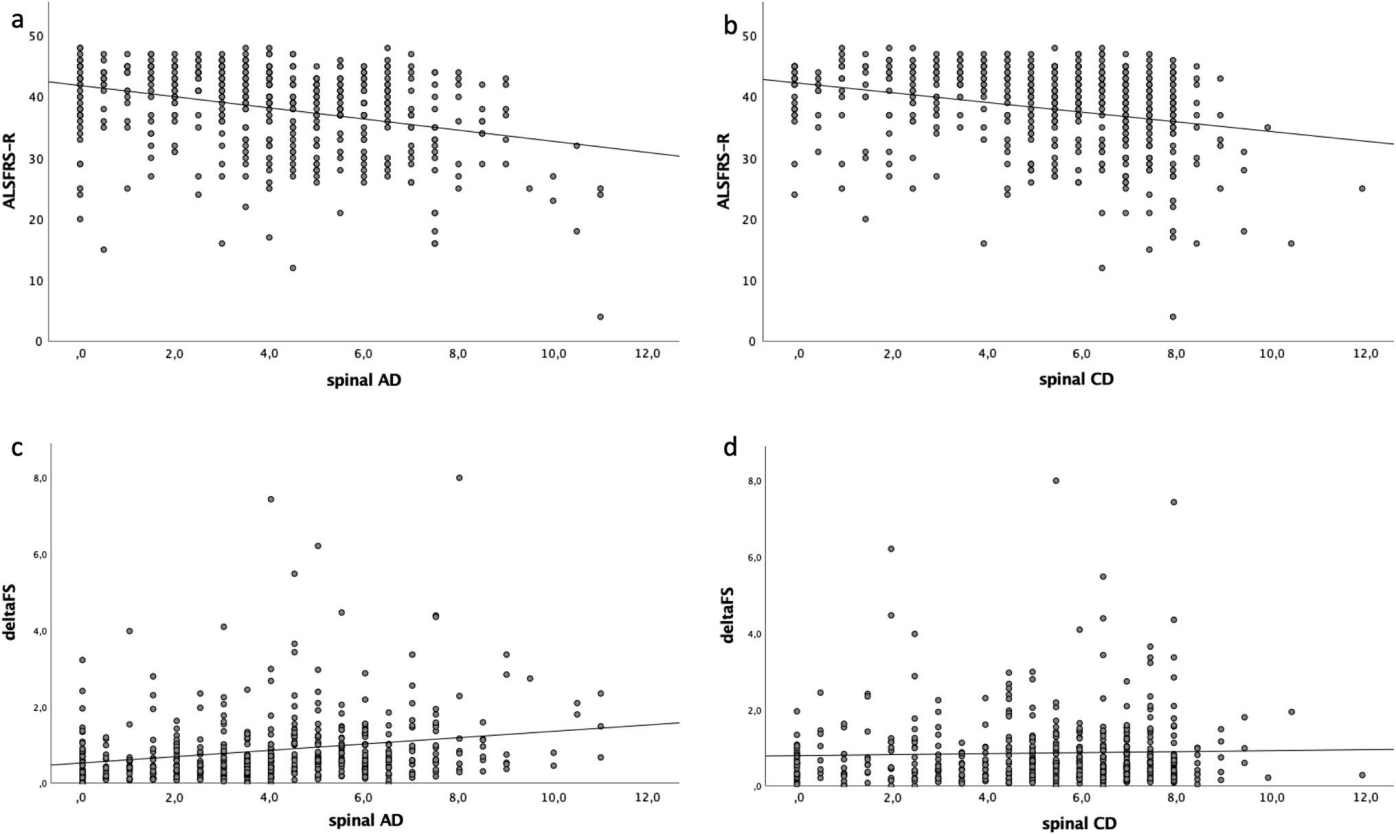
Scatter plots showing the correlation of ALSFRS with spinal AD (*R*^2^ = 0.105, *p = *3.17 × 10^–13^) and CD (*R*^2^ = 0.078, *p = *3.6 × 10^–5^) scores (**a** and **b**) and of ΔFS with spinal AD (*R*^2^ = 0.049 *p = *1.0 × 10^–6^) and CD (*R*^2^ = 0.001 *p = *0.46) scores (**c** and **d**). Each grey dot represents a single patient. Trend line is shown in black

We then evaluated the association between denervation scores to ALSFRS-R, ΔFS, disease stage and survival to define if EMG findings represent a reliable tool for assessing functional disability, disease progression and prognosis. We found that patients with lower ALSFRS-R values had significantly higher AD scores in the spinal region (*R*^2^ = 0.105, *p = *3.17 × 10^–13^), and CD scores in both the spinal (*R*^2^ = 0.078, *p = *3.6 × 10^–5^) and bulbar (*R*^2^ = 0.039, *p = *4.32 × 10^–10^) segments (Fig. 2a, b), suggesting that EMG signs of LMN dysfunction predict functional disability in ALS.

With regard to ΔFS, we observed a direct correlation with AD in the spinal regions (*R*^2^ = 0.049 *p = *1.0 × 10^–6^) (Fig. 2c, d). Conversely, we did not find any association with CD parameters, suggesting that active denervation alone is a marker of disease progression rate.

**Fig. 3:**
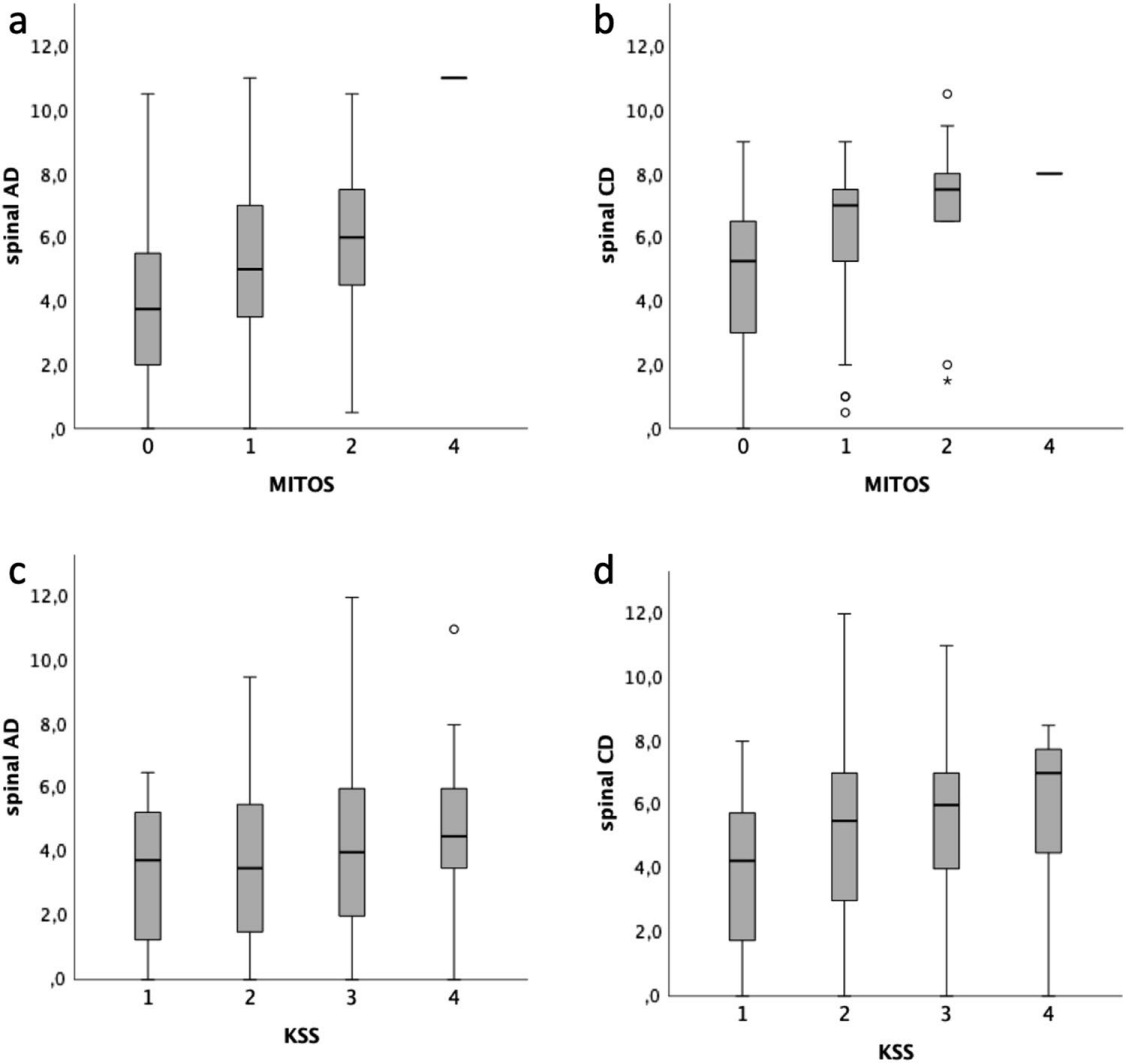
Correlation between MITOS stages and spinal AD (*p = *6.4 × 10^–7^) and CD (*p = *1.9 × 10^–7^) scores (**a** and **b**) and between King’s stages (KSS) and spinal AD (*p = *0.025) and CD (*p = *0.001) scores (**c** and **d**). For each group the bold line shows the median, the grey boxes includes the middle 50% of the data and whiskers show the minimum and maximum values. Empty circles and asterisks represent outliers. Kruskal–Wallis test for independent samples

These results were also supported by the findings regarding MITOS and KSS staging systems. Indeed, we observed an association between more severe MITOS stages with higher spinal AD (*p = *6.4 × 10^–7^) and CD (*p = *1.9 × 10^–7^) scores (Fig. 3a, b). In particular, patients at stages 1 and 2 had a significantly higher burden of active and chronic denervation signs compared to stage 0 [stage 1 vs 0: (AD: *p = *4.0 × 10^–6^; CD: *p = *3.0 × 10^–6^); stage 2 vs 0 (AD: *p = *0.002; CD: *p = *2.6 × 10^–4^)]. We found a similar association with regard to KSS stages for spinal AD (*p = *0.025) and CD (*p = *0.001) scores (Fig. 3c, d), as well as for bulbar CD score (*p = *0.004).

**Fig. 4:**
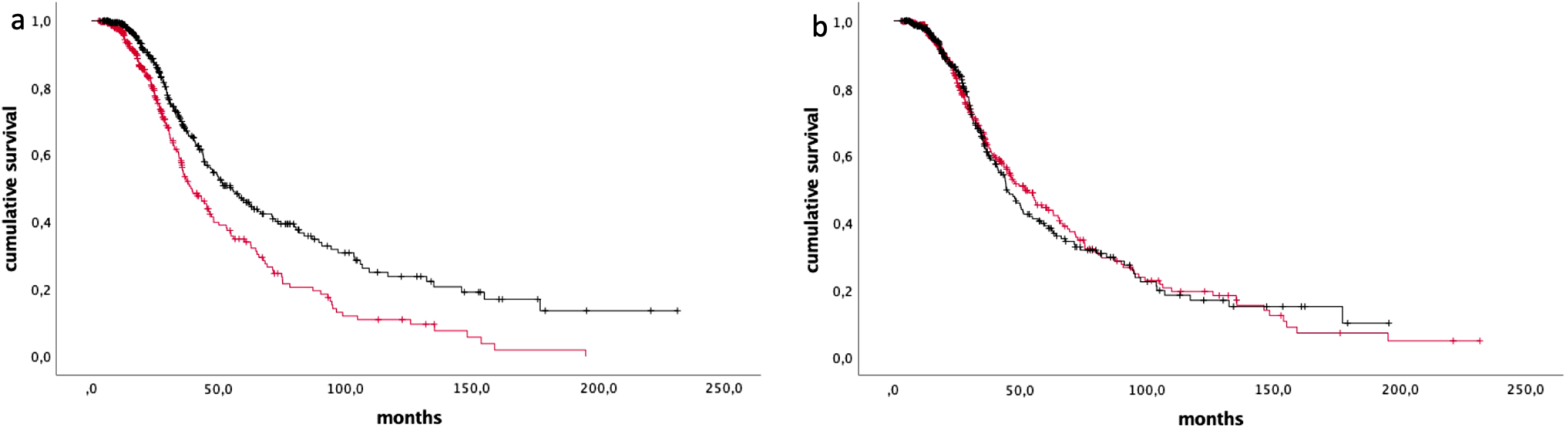
Kaplan–Meier plots of survival probabilities for AD (*p = *1.1 × 10^–5^) (**a**) and CD (*p = *0.86) (**b**). Red line: patients with values above the median; black line: patients with values below the median (median values: 4.0 for AD, 5.5 for CD). + : censored cases

Notably, after subdividing our cohort in two groups, according to the median value of spinal AD (4.0), we found that patients with less severe signs of active denervation had significantly longer survival (41.7 ± 40.2 vs 32.3 ± 29.0, *p = *1.1 × 10^–5^) (Fig. 4a). Conversely, we did not appreciate any association between survival and CD scores (median 5.5) (39.8 ± 37.6 vs 35.4 ± 34.3, *p = *0.86 (Fig. 4b). At multivariate analysis, using as covariates gender, age at onset, site of onset and *C9orf72* status, higher spinal AD scores were an independent predictor of shorter survival [HR = 1.2 (95% CI = 1.1–1.2), *p = *7.3 × 10^–9^], while no effect was observed for spinal CD [HR = 1.0 (95% CI = 0.9–1.1), *p = *0.273]. Similarly, bulbar AD scores correlated with shorter survival [HR = 1.7 (95% CI = 1.3–2.4), *p = *5.9 × 10^–4^], while bulbar CD did not [HR = 1.0 (95% CI = 0.8–1.2), *p = *0.854]. Lastly, no association was identified for AD and CD scores with age at onset.

## Discussion

EMG represents the most important diagnostic test in ALS as it can confirm the presence of muscle weakness due to anterior horn cell loss and it can reveal LMN involvement in clinically silent segments. As such, following the Awaji criteria, neurophysiological evidence of LMN dysfunction has now equivalent weight to clinical manifestations. Nevertheless, although EMG is almost always performed to confirm the clinical suspicion in ALS patients, there is relatively little information about its prognostic role. For these reasons, we investigated the correlation between neurophysiological signs of active and chronic denervation with clinical features, progression rate, and survival in a large cohort of ALS patients. We analysed the role of these EMG parameters as AD can be considered a marker of an ongoing muscle damage and CD an indicator of previously occurred muscle injury.

We found that patients with higher spinal AD and CD values showed significantly lower MRC scores and higher LMN scores. The association between these EMG parameters and clinical signs of LMN involvement suggests that AD and CD can be not only reliable biomarkers of muscle weakness [11] but also good predictors of the burden of clinical LMN signs. Furthermore, results obtained from correlations of AD and CD with ALSFRS-R underline the importance of these EMG findings as markers of functional disability in ALS individuals.

We observed a more rapid disease progression, as measured by the ΔFS, in those subjects with higher AD scores, while no such phenomenon could be observed for CD, suggesting that only active denervation signs are a good predictor of disease progression. In literature, previous similar investigations have been performed. For example, one report [12] studied the correlation of denervation signs (both active and chronic) in bulbar, cervical and lumbosacral regions with the progression from mild to severe ALS forms and with the deterioration of daily life activities (based on loss of speech function, loss of upper limb function, and loss of walking ability). This report, analysing a cohort of 363 patients, found that active denervation findings (defined as fibrillation potentials or positive sharp waves) in the cervical-upper limb area were prognostic factors for progression from mild to severe disability. In another study on 31 ALS patients, a higher occurrence of multiple discharges at baseline appeared to correlate with greater decline of the ALSFRS-R score, although this was not statistically significant [18]. In this cohort, ALS patients with multiple discharges at baseline showed deterioration in the fine motor function ALSFRS-R sub scores (items 4 and 5) between visits 10 weeks apart, while stability was observed in individuals without this EMG finding. Another report evaluated the relationship between high-density motor unit number estimation (MUNE) and ALSFRS-R in monitoring disease progression [19]. Patients classified as rapid progressors according to the decrement in MUNE at 4-month follow-up showed significantly lower ALSFRS-R scores at 8-month follow-up. In that study, MUNE proved to be more sensitive to motor neuron loss early in the disease course when compared to other clinical measures.

AD and CD were also directly correlated with MITOS and KSS, supporting the role of EMG parameters as markers of disease stage. Moreover, with regard to results obtained from survival analysis, we found an inverse correlation with AD, both in bulbar and spinal regions, indicating the importance of EMG as a prognostic instrument. Furthermore, our study underlined the major role of the presence of an ongoing active muscle damage (AD) in predicting survival if compared to the presence of previously remodelled motor units (CD). Indeed, notably, CD well correlates with ALS staging system scores and functional disability but is not associated with survival. Similarly, other previous studies analysed the role of EMG as a prognostic instrument.

A recent study of 92 patients calculated denervation values, considering for the score only sites that showed the contemporary presence of active and chronic denervation signs, for bulbar, cervical and lumbosacral regions. In this report higher general denervation scores (calculated summing the scores of the three body regions) and higher bulbar denervation scores were significantly associated with a reduced survival [11]. In another report of 103 patients, neurophysiological genioglossus involvement (defined by the contemporary presence of active and chronic denervation signs) at diagnosis was associated with reduced survival, a shorter time to moderate dysphagia and to severe dysarthria and it was a stronger prognostic factor than the presence of clinical bulbar lower motor neuron signs alone [12].

Our study has some limitations. Firstly, our cohort includes a relatively small number of patients at more advanced disease stages. Additionally, ours is a cross-sectional study and therefore longitudinal data on independent cohorts will be needed to assess evolution of EMG parameters over the course of the disease. The relatively low number of muscles studied does not allow to correlate specific EMG regional patterns with ALS phenotype and prognosis. We also did not routinely perform EMG of thoraco-abdominal muscles, and thus we cannot draw any association between neurophysiological parameters and respiratory function in our patients. Finally, the calculation of AD and CD scores was performed by a single neurophysiologist with experience in the field of motor neuron disease. As such, although the evaluator was blinded to clinical phenotype of ALS patients, it is not possible to determine the inter-rater reliability of AD and CD scores. Conversely, the EMG protocol adopted in our study is reproducible, being routinely used in a clinical setting at our ALS Clinic and generally well-tolerated by patients.

Our results confirm that EMG examination represents not only an essential diagnostic instrument, but also an important tool to better characterise phenotype, functional disability, disease progression and prognosis in ALS. Furthermore, we investigated the different role of AD, which in our work seems to be a reliable predictor of disease progression and survival, and CD, which conversely seems to better describe functional disability. Ours is the first study that describes all these correlations in a large cohort of patients, although longitudinal data on independent ALS populations will be needed to confirm these results.

## Supplementary Information

Below is the link to the electronic supplementary material.

Supplementary file1 (DOCX 13 kb)
