# Correction to: Correlation between clinical phenotype and electromyographic parameters in amyotrophic lateral sclerosis

**DOI:** 10.1007/s00415-023-11652-y

**Published:** 2023-03-13

**Authors:** Eleonora Colombo, Alberto Doretti, Francesco Scheveger, Alessio Maranzano, Giulia Pata, Delia Gagliardi, Megi Meneri, Stefano Messina, Federico Verde, Claudia Morelli, Stefania Corti, Luca Maderna, Vincenzo Silani, Nicola Ticozzi

**Affiliations:** 1 Department of Neurology and Laboratory of Neuroscience, IRCCS Istituto Auxologico Italiano, P.le Brescia 20, 20149 Milan, Italy; 2 Neurology Residency Program, Università degli Studi di Milano, Via Festa del Perdono 7, 20122 Milan, Italy; 3 Faculty of Medicine and Surgery, Università degli Studi di Milano, Via Festa del Perdono 7, 20122 Milan, Italy; 4 Neurology Unit, Fondazione IRCCS Ca’ Granda Ospedale Maggiore Policlinico, Via Francesco Sforza 35, 20122 Milan, Italy; 5 Department of Pathophysiology and Transplantation, Università degli Studi di Milano, Via Festa del Perdono 7, 20122 Milan, Italy

**Correction to: Journal of Neurology (2023) 270:511–518** 10.1007/s00415-022-11404-4

The original version of this article unfortunately contained a mistake. There are corrections in the affiliations of the authors.

Original Affiliations

Eleonora Colombo^1^ · Alberto Doretti^1^ · Francesco Scheveger^1,2^ · Alessio Maranzano^1,2^ · Giulia Pata^1^ ·Delia Gagliardi^3^ · Megi Meneri^3,4^ · Stefano Messina^1^ · Federico Verde^1,4^ · Claudia Morelli^1^ · Stefania Corti^3,4^ · Luca Maderna^1^ · Vincenzo Silani^1,4^ · Nicola Ticozzi^1,4^

- ^1^Department of Neurology and Laboratory of Neuroscience, IRCCS Istituto Auxologico Italiano, P.le Brescia 20, 20149 Milan, Italy
- ^2^Neurology Residency Program, Università degli Studi di Milano, Via Festa del Perdono 7, 20122 Milan, Italy
- ^3^Neurology Unit, Fondazione IRCCS Ca’ Granda Ospedale Maggiore Policlinico, Via Francesco Sforza 35, 20122 Milan, Italy
- ^4^Department of Pathophysiology and Transplantation, Università degli Studi di Milano, Via Festa del Perdono 7, 20122 Milan, Italy

Correct Affiliations

Eleonora Colombo^1^ · Alberto Doretti^1^ · Francesco Scheveger^2^ · Alessio Maranzano^2^ · Giulia Pata^3^ ·Delia Gagliardi^4^ · Megi Meneri^4,5^ · Stefano Messina^1^ · Federico Verde^1,5^ · Claudia Morelli^1^ · Stefania Corti^4,5^ · Luca Maderna^1^ · Vincenzo Silani^1,5^ · Nicola Ticozzi^1,5^

- ^1^Department of Neurology and Laboratory of Neuroscience, IRCCS Istituto Auxologico Italiano, P.le Brescia 20, 20149 Milan, Italy
- ^2^Neurology Residency Program, Università degli Studi di Milano, Via Festa del Perdono 7, 20122 Milan, Italy
- ^3^Faculty of Medicine and Surgery, Università degli Studi di Milano, Via Festa del Perdono 7, 20122 Milan, Italy
- ^4^Neurology Unit, Fondazione IRCCS Ca’ Granda Ospedale Maggiore Policlinico, Via Francesco Sforza 35, 20122 Milan, Italy
- ^5^Department of Pathophysiology and Transplantation, Università degli Studi di Milano, Via Festa del Perdono 7, 20122 Milan, Italy

