# Supplementary material for: Correlation between clinical phenotype and electromyographic parameters in amyotrophic lateral sclerosis
Source: J Neurol. 2022 Oct 2;270(1):511–8. doi: 10.1007/s00415-022-11404-4 (PMC9813173; doi:10.1007/s00415-022-11404-4)
Supplement: Supplementary file 1 — Supplementary file1 (DOCX 13 kb) [file 415_2022_11404_MOESM1_ESM.docx]

**Supplemental table 1**: criteria used to calculate active (AD) and chronic denervation (CD) scores for the bulbar region (right genioglossus, left and right masseter), right arm (biceps brachii, first dorsal interosseus), left arm (triceps brachii, first dorsal interosseus), right lower limb (vastus medialis, tibialis anterior) and left lower limb (gastrocnemius medialis, tibialis anterior).

* in case of different scores between muscles, the highest score was considered.
** in case of expression such as “slightly”, “mainly” or in the presence of alternatives (for example “normal or increased duration”), we approximated the score upwards. In case of muscles with different scores, an arithmetic average was made

| ***Active denervation***  **(spontaneus activity)*** | ***Chronic denervation***  **(MUAPs characteristics)**** | ***Score*** |
| --- | --- | --- |
| absent | Normal amplitude and duration | 0 |
| Fibrillation potentials and positive sharp waves +  *or*  insertional activity +  *or*  Presence of high frequency discharge  *or*  fibrillation potentials +/- | Increased duration, normal amplitude  *or*  normal duration, increased amplitude  *or*  decreased amplitude, increased/ decreased/normal duration  *or*  decreased duration, normal amplitude | 1 |
| Fibrillation potentials and positive sharp waves ++ | Increased duration, increased amplitude  *or*  very increased duration and amplitude | 2 |
| Fibrillation potentials and positive sharp waves +++ *or* ++++  *or*  Fibrillation potentials and positive sharp waves in every site | No activity during voluntary muscle activation | 3 |

Abbreviations: MUAPS= Motor Unit Action Potentials
